# Identifying gene expression signatures for risk stratification of postoperative adjuvant chemotherapy in colorectal cancer

**DOI:** 10.1002/2211-5463.70243

**Published:** 2026-03-30

**Authors:** Mayuko Otomo, Keisuke Okuno, Shuichi Watanabe, Sakiko Oba, Hiroyasu Kagawa, Masanori Tokunaga, Daisuke Ban, Yusuke Kinugasa

**Affiliations:** ^1^ Department of Gastrointestinal Surgery Institute of Science Tokyo Japan; ^2^ Division of Cellular Signaling National Cancer Center Research Institute Tokyo Japan; ^3^ Department of Hepatobiliary and Pancreatic Surgery Institute of Science Tokyo Japan

**Keywords:** adjuvant chemotherapy, colorectal cancer, gene expression, prognostic biomarker, recurrence prediction, risk stratification

## Abstract

Clinical risk stratification for postoperative recurrence in patients with pathological stage II (pStage II) colorectal cancer (CRC) is essential for guiding the use of postoperative adjuvant chemotherapy (ACT). In this study, we identified novel prognostic gene expression biomarkers in patients with pStage II CRC and developed a new risk stratification framework for ACT decision‐making. First, genome‐wide biomarker discovery was conducted to identify prognostic gene expression biomarkers associated with recurrence risk in pStage II CRC. This analysis identified 10 differentially expressed genes as potential biomarkers for recurrence. The efficacy of these biomarkers was then tested using 188 clinical surgical specimens obtained from patients with pStage II CRC. A predictive panel was developed using qRT‐PCR and used to assess 93 clinical specimens with an area under the curve (AUC) of 0.82, and its performance was further validated in an independent cohort (*n* = 95). By incorporating key clinicopathological features, a Gene expression‐based Prediction of Recurrence in pStage II CRC (GPRSC) signature was developed, which robustly predicted postoperative recurrence (AUC: 0.80). Finally, combining the GPRSC signature, microsatellite instability status, and conventional criteria, we developed a novel risk stratification system for postoperative ACT decision‐making in pStage II CRC. Overall, we identified novel gene expression biomarkers and developed a prognostic signature that informs clinical decision‐making regarding postoperative ACT in patients with pStage II CRC.

## Graphical abstract

A novel signature integrating genome‐wide analysis with clinical factors predicts recurrence in stage II colorectal cancer and enables a new risk stratification to guide postoperative adjuvant chemotherapy.

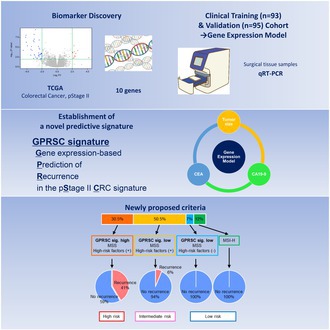

**Abbreviations**
ACT: adjuvant chemotherapy
AUC: area under the curve
CA19‐9: carbohydrate antigen 19–9
cDNA: complementary DNA
CEA: carcinoembryonic antigen
CI: confidence interval
CRC: colorectal cancer
ctDNA: circulating tumor DNA
DSS: disease‐specific survival
FC: fold‐change
GPRSC: Gene expression‐based prediction of recurrence in pStage II CRC
HR: hazard ratio
JSCCR guidelines: Japanese Society for Cancer of the Colon and Rectum guidelines for the treatment of CRC
LASSO: least absolute shrinkage and selection operator
MSI‐H: microsatellite instability high
MSS: microsatellite stable
pStage: pathological stage
qRT‐PCR: quantitative reverse transcription polymerase chain reaction
RFS: recurrence‐free survival
ROC: receiver operating characteristic
TCGA: The Cancer Genome Atlas

Surgical resection of tumors is the standard treatment for patients with pathological stage (pStage) II and III colorectal cancer (CRC). To reduce the risk of recurrence, postoperative adjuvant chemotherapy (ACT) is recommended following radical surgery of the primary tumors in patients with pStage III and high‐risk pStage II CRC. This recommendation is supported by guidelines from organizations, including the NCCN, ASCO, and ESMO [1, 2, 3]. Nevertheless, in view of survival benefits, toxicity, and medical costs, ongoing research continues to investigate the optimal regimen and duration of ACT. Clinical decision‐making for postoperative ACT, including the selection of appropriate patient subgroups and optimal treatment regimens, remains a major challenge in managing patients with stage II and III CRC [4, 5]. In this context, several prospective clinical trials, including the IDEA collaboration and TOSCA trials, have been conducted to investigate the survival noninferiority and potential benefits in adverse events of shortening the duration of postoperative ACT in patients with pStage II and III CRC [6, 7, 8], yet the results remain controversial. In addition, several secondary studies using clinical trial specimens have identified predictive molecular biomarkers for the efficacy of postoperative ACT in these patients [9, 10, 11, 12]; nonetheless, none of these molecular biomarkers have been applied in clinical practice.

In patients with pStage II CRC, postoperative ACT is recommended for those with high‐risk factors of recurrence [1, 2, 3]. These high‐risk factors include pathological and clinical tumor features such as T4, poor differentiation, lymphovascular invasion, perineural invasion, high tumor budding scores, inadequate lymph node harvest (<12), clinical obstruction, and perforation. However, the existing definition of high‐risk pStage II disease is inadequate because a considerable number of patients do not experience recurrence [13, 14]. Therefore, several studies have sought to identify a subset of patients who are at a high risk of recurrence and who will benefit from postoperative ACT by analyzing their clinical and molecular features. As a prognostic molecular feature, pStage II CRC patients with microsatellite instability high (MSI‐H) status typically reveal a more favorable prognosis than those with microsatellite stable (MSS) CRCs, and no significant benefit from postoperative ACT has been demonstrated for those patients [15, 16]. In addition, the presence of RAS and BRAF mutations in stage II CRC is associated with poor prognosis. These mutations confer resistance to anti‐EGFR monoclonal antibodies, rendering such therapies ineffective, and are not indicated for patients harboring these genetic alterations [16]. Furthermore, circulating tumor DNA (ctDNA)‐based assessment of molecular residual disease, immunoscore, and multigene expression assays have demonstrated clinical utility in various studies, including appropriate selection of candidates for postoperative ACT, optimization of treatment duration, early detection of recurrence, and monitoring of therapeutic efficacy [17, 18, 19]. However, except for MSI status, no definitive molecular biomarkers are routinely used in clinical practice.

In this study, through comprehensive biomarker discovery, we identified novel gene expression‐based biomarkers for predicting tumor recurrence in patients with pStage II CRC. Thereafter, we used quantitative reverse transcription polymerase chain reaction (qRT‐PCR) assay to perform biomarker training and validation on clinical surgical specimens, generating a gene expression‐based predictive signature for recurrence in patients with pStage II CRC. Finally, by integrating these biomarkers with existing risk classification systems, we developed a novel framework for the risk stratification of postoperative ACT in patients with pStage II CRC.

## Methods

### Comprehensive gene expression‐based biomarker discovery

To identify candidate gene expression‐based biomarkers for predicting recurrence in patients with pStage II CRC, we analyzed The Cancer Genome Atlas (TCGA) level 3 RNA sequencing dataset. TCGA dataset was downloaded from the University of California Santa Cruz Xena Browser (https://xenabrowser.net/) and included 128 patients with pStage II CRC (recurrence, *n* = 31; non recurrence, *n* = 97). The clinicopathological characteristics of the patients are summarized in Table S1. We used DESeq2 to identify differentially expressed genes between the recurrence and nonrecurrence groups (|Log_2_ fold change [FC]| > 2.0, *P* < 0.01) [20], and the least absolute shrinkage and selection operator (LASSO) analysis was performed for variable selection and estimation of regression coefficients [21].

### Clinical patient cohorts

This study included a total of 188 clinical surgical specimens from patients with pStage II CRC who underwent curative surgery at the Institute of Science Tokyo Hospital between 2012 and 2018. All patients underwent curative resection for primary CRC following the Japanese Society for Cancer of the Colon and Rectum guidelines for the treatment of CRC (JSCCR guidelines) [22], and no patient in this cohort received ACT after curative resection of primary tumors. All tumors were histologically diagnosed as pStage II by experienced pathologists, according to the TNM Classification of Malignant Tumors (UICC 8th edition). Between 2012 and 2015, 93 patients were assigned to the clinical training cohort, whereas 95 enrolled between 2016 and 2018 comprised the clinical validation cohort. The surgical specimens were divided into two cohorts based on the collection period to ensure temporal independence between the two cohorts and to mimic real‐world external validation. We also confirmed that the clinicopathological characteristics were comparable between the two cohorts. The clinicopathological characteristics of each cohort are shown in Table 1.

**Table 1:** Clinicopathological characteristics of clinical cohorts. Categorical variables were compared using Fisher's exact test, whereas continuous variables were analyzed using a two‐tailed Student's *t*‐test. SD, standard deviation; CEA, carcinoembryonic antigen; CA19‐9, carbohydrate antigen 19–9.

		Clinical training cohort (*n* = 93)		Clinical validation cohort (*n* = 95)		*P* value
		*n*	(%)	*n*	(%)	
Age (years)	Mean (±SD)	70	(±11)	70	(±11)	0.91
Sex	Male	57	(61)	58	(61)	1.00
	Female	36	(39)	37	(39)	
Tumor location	Right‐sided	27	(29)	32	(34)	0.53
	Left‐sided	66	(71)	63	(66)	
Tumor size (mm)	Mean (±SD)	49	(±24)	49	(±21)	0.97
Tumor depth	T3	80	(86)	78	(82)	0.55
	T4	13	(14)	17	(18)	
Lymphatic invasion	Positive	16	(17)	15	(16)	0.85
	Negative	77	(83)	80	(84)	
Vascular invasion	Positive	76	(82)	81	(85)	0.56
	Negative	17	(18)	14	(15)	
CEA	> 5.0 ng·mL^−1^	38	(41)	40	(42)	0.88
	≤ 5.0 ng·mL^−1^	55	(59)	55	(58)	
CA19‐9	> 37 U·mL^−1^	7	(8)	6	(6)	0.78
	≤ 37 U·mL^−1^	86	(92)	89	(94)	

Clinical data, including demographics, comorbidities, recurrence, and survival outcomes, were collected from electronic medical records. Follow‐up was continued until death or for at least 5 years postoperatively in accordance with JSCCR guidelines [22]. Written informed consent was obtained from all patients. This study was approved by the Ethics Committee of the Institute of Science Tokyo Hospital (approval no.: M2000‐831) and conducted in accordance with the Declaration of Helsinki.

### RNA extraction and qRT‐PCR assays

Total RNA was extracted from frozen surgical specimens using the RNeasy Mini Kit (QIAGEN, Hilden, Germany), and complementary DNA (cDNA) was synthesized using the High‐Capacity cDNA Reverse Transcription Kit (Thermo Fisher Scientific, Waltham, MA, USA). qRT‐PCR was performed using the SensiFAST SYBR Lo‐ROX Kit (Bioline, London, UK) on a StepOnePlus Real‐Time PCR System (Applied Biosystems, Foster City, CA, USA) as described previously [23]. Relative gene expression was calculated using the 2−ΔCt method, with Beta Actin as the internal control. Primer sequences were obtained from PrimerBank (https://pga.mgh.harvard.edu/primerbank/). The specificity of each primer pair was confirmed by BLAST analysis against the GenBank database to ensure target‐specific amplification and to minimize nonspecific binding. Primer sequences used in this study are listed in Table S2.

### Genomic DNA extraction and MSI analyses

Genomic DNA was extracted from frozen surgical specimens using the RNeasy Mini Kit (QIAGEN). MSI analyses were conducted using an ABI 3130 Genetic Analyzer (Applied Biosystems) as described previously [24, 25]. Five mononucleotide repeat markers (BAT25, BAT26, D2S123, D5S346, and D17S250) were assessed. According to the Bethesda Guidelines established by the National Cancer Institute, tumors were classified as MSI‐H if instability was detected in two or more of the five markers. Tumors without unstable markers were defined as those with MSS.

### Statistical analysis

All statistical analyses were performed using EZR version 1.68, which is a graphical user interface for R (R Foundation for Statistical Computing, Vienna, Austria, version 4.3.1) [26]. Categorical variables were compared using Fisher's exact test, whereas continuous variables were analyzed using a two‐tailed Student's *t*‐test. The cutoff values for continuous variables were determined based on the mean values within each clinical cohort. Survival curves were generated using the Kaplan–Meier method and compared using the log‐rank test. A predictive signature based on gene expression level was developed using logistic regression analysis. Model performance was assessed using receiver operating characteristic (ROC) curves as well as the area under the curve (AUC). The optimal cutoff threshold was determined by maximizing the Youden index (sensitivity + specificity − 1) derived from the ROC curve analysis. Statistical significance was defined as a two‐sided *P* value of < 0.05.

## Results

### Identification of 10 novel candidate gene expression‐based biomarkers for prediction of recurrence in pStage II CRC

**Fig. 1:**
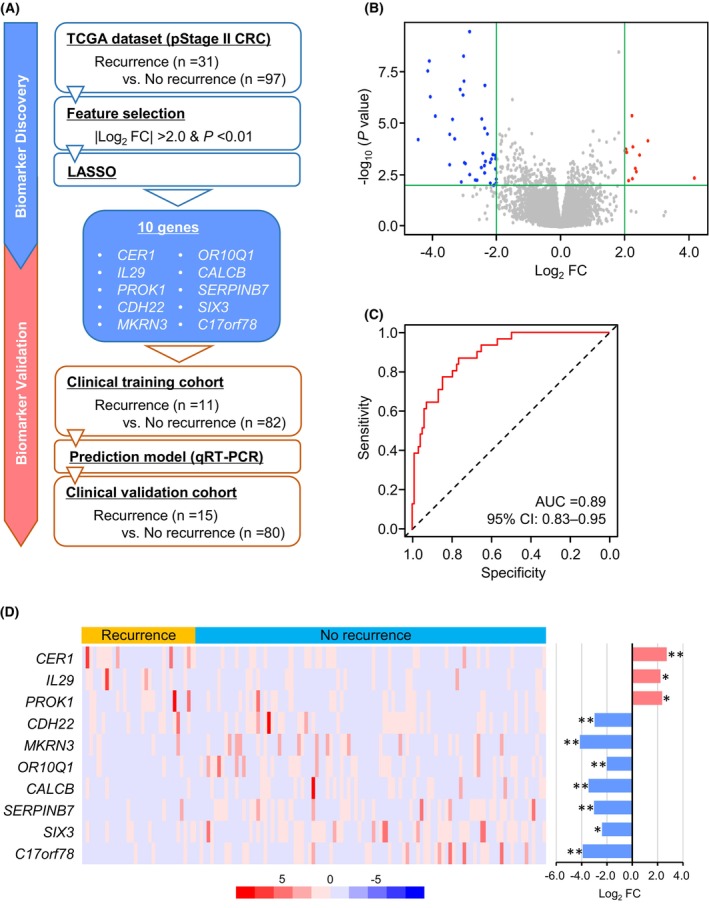
Biomarker discovery phase for the candidate genes to predict recurrence in patients with pStage II CRC. (A) Schematic of the study design. (B) Volcano plot of genes for predicting recurrence in TCGA dataset. The red and blue dots represent significantly up‐regulated (Log_2_ FC > 2.0 and *P* < 0.01) and down‐regulated (Log_2_ FC < –2.0 and *P* < 0.01) genes in patients with recurrence, respectively. (C) ROC curve values for the 10‐gene panel in TCGA dataset. AUC and 95% CI were calculated using the DeLong method. (D) Heatmap of 10 candidate genes in TCGA dataset. *P < 0.01, **P < 0.001. TCGA, The Cancer Genome Atlas; CRC, colorectal cancer; pStage, pathological stage; FC, fold change; LASSO, least absolute shrinkage and selection operator; qRT‐PCR, quantitative reverse transcription polymerase chain reaction; ROC, receiver operating characteristics; AUC, area under the curve; CI, confidence interval.

An overview of the study design is presented in Fig. 1A. To identify candidate biomarkers for predicting recurrence in patients with pStage II CRC, we analyzed TCGA RNA sequencing dataset, which included 128 patients with pStage II CRC. In this analysis, 52 genes were significantly differentially expressed in patients with recurrence compared to those without recurrence (|Log_2_ FC| > 2.0, *P* < 0.01; Fig. 1B and Table S3). Subsequently, LASSO analysis was performed to refine the list of candidate biomarkers, and 10 genes—*CER1*, *IL29*, *PROK1*, *CDH22*, *MKRN3*, *OR10Q1*, *CALCB*, *SERPINB7*, *SIX3*, and *C17orf78—*were identified as candidate biomarkers for predicting recurrence in patients with pStage II CRC. In TCGA dataset, the logistic regression panel using these 10 genes revealed a promising predictive potential with an AUC value of 0.89 (95% confidence interval [CI]: 0.83–0.95; Fig. 1C). The details of the expression of these 10 genes are presented in Fig. 1D. Overall, by analyzing a genome‐wide dataset, we identified 10 novel gene expression‐based biomarkers for predicting recurrence in patients with pStage II CRC.

### Establishment of gene expression panel comprising 10 genes for predicting recurrence in clinical training cohort

**Fig. 2:**
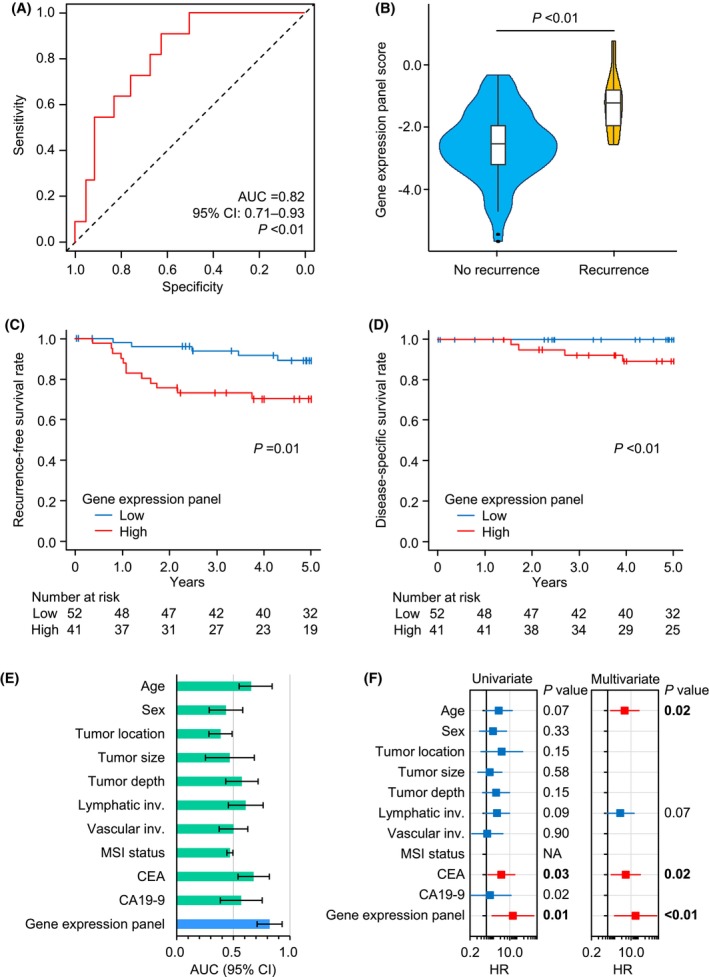
Clinical training phase for the gene expression panel to predict recurrence in patients with pStage II CRC. (A) ROC curve values for the gene expression panel in clinical training cohort. AUC, 95% CI, and *P* value were calculated using the DeLong method. (B) Violin plot for the gene expression panel score in patients with recurrence and without recurrence. The central line indicates the median, and the box (bar) represents the interquartile range. The width of the violin represents the data density. Statistical comparisons were performed using the Student's *t*‐test. (C, D) Kaplan–Meier curve for the recurrence‐free survival (C) and the disease‐specific survival (D) for the patient with low and high gene expression panel score in clinical training cohort. *P* values were calculated using the log‐rank test. (E) Bar graph with AUC values of key clinical features and gene expression panel in clinical training cohort. Error bars indicate 95% CI. (F) Forest plot with HR of key clinical features and gene expression panel in clinical training cohort. HR with 95% CI and *P* value were calculated using a multivariate Cox proportional hazards model. Error bars represent 95% CI. CRC, colorectal cancer; ROC, receiver operating characteristics; AUC, area under the curve; CI, confidence interval; MSI, microsatellite instability; CEA, carcinoembryonic antigen; CA19‐9, carbohydrate antigen 19–9; HR, hazard ratio.

To evaluate the prognostic potential of the candidate biomarkers in clinical specimens, we performed qRT‐PCR using total RNA extracted from surgical tissue samples using a clinical training cohort. Overall, 93 patients (recurrence, *n* = 11; non recurrence, *n* = 82) were enrolled in the clinical training cohort (Table 1), which included 5 MSI‐H cases (5.4%; Fig. S1). Using logistic regression analysis, we generated a gene expression panel comprising 10 genes to predict recurrence in patients with pStage II CRC. The scoring formula was as follows: Logit(P) = (0.44896 × *CER1*) + (0.01347 × *IL29*) + (0.71132 × *PROK1*) + (−0.84953 × *CDH22*) + (0.10031 × *MKRN3*) + (−1.07897 × *OR10Q1*) + (1.05113 × *CALCB*) + (−1.04955 × *SERPINB7*) + (0.92483 × *SIX3*) + (0.12277 × *C17orf78*) + 0.97488. These coefficients of the scoring formula were derived using multivariable logistic regression analysis. The expression levels of the selected genes were entered into the model as continuous variables, and regression coefficients were estimated by the maximum likelihood method. This panel yielded a robust predictive potential, with an AUC value of 0.82 (95% CI: 0.71–0.93; *P* < 0.01; Fig. 2A). A significant difference in the gene expression panel scores was observed between the recurrent and nonrecurrent groups (*P* < 0.01; Fig. 2B).

Next, patients in the clinical training cohort were stratified into high‐ and low‐score groups based on the cutoff value derived from the Youden index. In Kaplan–Meier analysis, the high‐score group revealed significantly worse 5‐year recurrence‐free survival (RFS) rate (70.3% vs. 89.1%; *P* = 0.01; Fig. 2C) and 5‐year disease‐specific survival (DSS) rate (89.4% vs. 100.0%; *P* < 0.01; Fig. 2D) than the low‐score group. Furthermore, this gene expression panel demonstrated superior AUC values compared with other key clinicopathological features in ROC curve analysis (Fig. 2E) and was a significant independent predictor for RFS in multivariate cox regression analysis (hazard ratio [HR]: 15.66; 95% CI: 1.96–125.40; *P* < 0.01; Fig. 2F). In summary, we successfully developed a gene expression panel consisting of 10 novel gene expression‐based biomarkers that can effectively predict recurrence in patients with pStage II CRC.

### Construction of gene expression model for enhanced predictive accuracy in clinical validation cohort

**Fig. 3:**
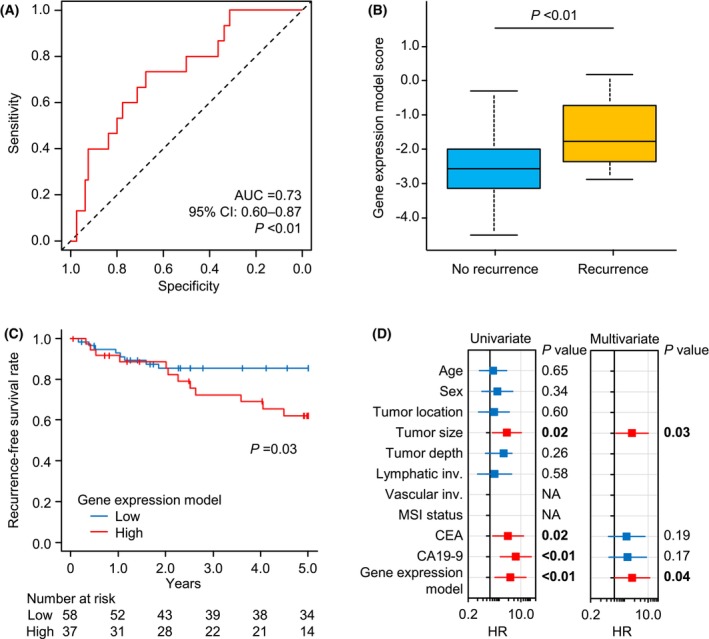
Clinical validation phase for the gene expression model to predict recurrence in patients with pStage II CRC. (A) ROC curve values for the gene expression model in the clinical validation cohort. AUC, 95% CI, and *P* value were calculated using the DeLong method. (B) Box plot for the gene expression model score in patients with and without recurrence. The central line indicates the median, and the box (bar) represents the interquartile range. Statistical comparisons were performed using the Student's *t*‐test. (C) Kaplan–Meier curve for the recurrence‐free survival for patients with low and high gene expression model score in the clinical validation cohort. *P* values were calculated using the log‐rank test. (D) Forest plot with HR of key clinical features and gene expression model in the clinical validation cohort. HR with 95% CI and *P* value were calculated using a multivariable Cox proportional hazards model. Error bars represent 95% CI. CRC, colorectal cancer; ROC, receiver operating characteristics; AUC, area under the curve; CI, confidence interval; MSI, microsatellite instability; CEA, carcinoembryonic antigen; CA19‐9, carbohydrate antigen 19–9; HR, hazard ratio.

Next, the predictive performance of the gene expression panel was validated using an independent cohort study. A total of 95 patients with pStage II CRC (recurrence, *n* = 15; non recurrence, *n* = 80) were enrolled in the clinical validation cohort. No significant differences were observed in the clinicopathological characteristics between the clinical training and validation cohorts (Table 1). Regarding MSI status, this cohort included 11 MSI‐H cases (11.6%; Fig. S1). The gene expression model could reasonably predict recurrence, with an AUC of 0.73 (95% CI: 0.60–0.87; *P* < 0.01; Fig. 3A). A significant difference in the gene expression model scores was observed between the recurrence and no‐recurrence groups (*P* < 0.01; Fig. 3B). Similar to the clinical training cohort, the high‐scoring group had a significantly worse 5‐year RFS rate than the low‐scoring group (62.4% vs. 85.5%; *P* = 0.03; Fig. 3C). In addition, in the univariate cox regression analysis, our gene expression model (HR: 4.62; 95% CI: 1.47–14.52; *P* < 0.01), tumor size (HR: 3.55; 95% CI: 1.21–10.42; *P* = 0.02), carcinoembryonic antigen (CEA; HR: 3.85; 95% CI: 1.23–12.12; *P* = 0.02), and carbohydrate antigen 19–9 (CA19‐9; HR: 6.85; 95% CI: 2.17–21.61; *P* < 0.01) were significant predictors of RFS. In the multivariate analysis including all variables that were significant in the univariate analysis, the gene expression model (HR: 3.39; 95% CI: 1.02–11.25; *P* = 0.04) and tumor size (HR: 3.35; 95% CI: 1.11–10.13; *P* = 0.03) were significant independent predictors for RFS in patients with pStage II CRC (Fig. 3D). Collectively, we successfully validated a gene expression model that allowed reasonable prediction of recurrence in patients with pStage II CRC.

### Establishment of genomic signature combining gene expression model with key clinicopathological features for recurrence prediction in pStage II CRC

**Fig. 4:**
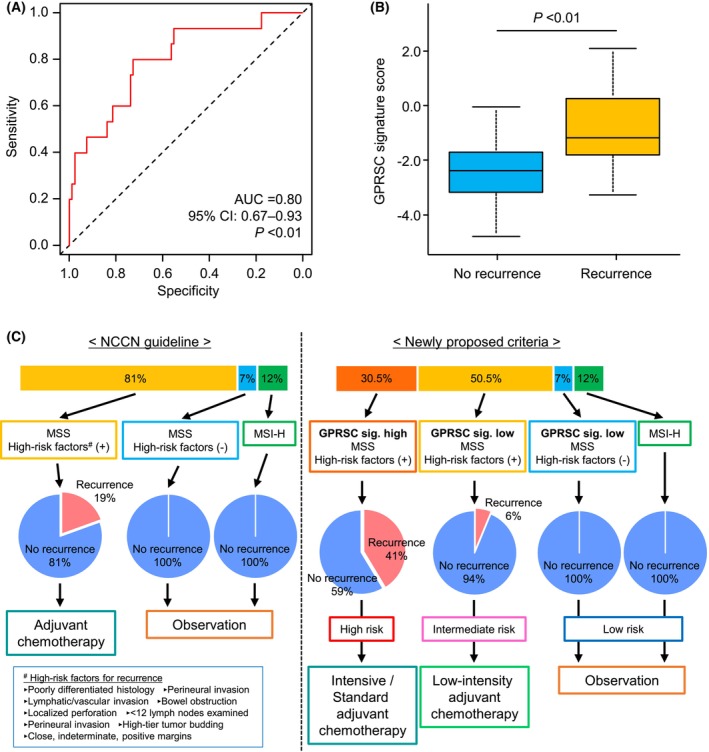
Establishment of GPRSC signature for the risk stratification of postoperative adjuvant chemotherapy in patients with pStage II CRC. (A) ROC curve values for the GPRSC signature in the clinical validation cohort. AUC, 95% CI, and *P* value were calculated using the DeLong method. (B) Box plot for the GPRSC signature score in patients with and without recurrence. The central line indicates the median, and the box (bar) represents the interquartile range. Statistical comparisons were performed using the Student's *t*‐test. (C) Comparison of postoperative treatment strategy between NCCN guidelines and newly proposed criteria using the GPRSC signature. CRC, colorectal cancer; ROC, receiver operating characteristic; AUC, area under the curve; CI, confidence interval; NCCN, National Comprehensive Cancer Network; MSI‐H, microsatellite instability high; MSS, microsatellite stable.

To improve the predictive performance of the gene expression model, we incorporated significant clinicopathological features (Fig. 3D), including tumor size, CEA, and CA19‐9, into our gene expression model and developed Gene expression‐based Prediction of Recurrence in the pStage II CRC (GPRSC) signature. This GPRSC signature demonstrated robust predictive performance for recurrence in a clinical validation cohort, with a corresponding AUC of 0.80 (95% CI: 0.67–0.93; *P* < 0.01; Fig. 4A). A significant difference in the GPRSC signature scores was observed between the groups with and without recurrence (*P* < 0.01; Fig. 4B).

To investigate the possible future clinical applications of our GPRSC signature, we compared the recurrence rate and recommended postoperative ACT treatment between the NCCN guideline [1] and GPRSC signatures (Fig. 4C). According to the NCCN guidelines, 81% of patients in the clinical validation cohort were categorized as having a high risk of recurrence (MSS tumor with at least one high‐risk factor) for whom postoperative ACT is recommended. Among them, only 19% experienced recurrence. In contrast, our novel GPRSC signature‐based strategy reclassified patients into three categories: (1) high risk: high GPRSC signature score and MSS tumors with high‐risk factors (30.5% of patients); (2) intermediate risk: low GPRSC signature score and MSS tumors with high‐risk factors (50.5% of patients); and (3) low risk: low GPRSC signature score and MSS without high‐risk factors (7.0% of patients) or MSI‐H tumors (12.0% of patients). In the high‐risk group categorized using our novel strategy, 41% of the patients experienced recurrence, suggesting the need for more intensive ACT, whereas only 6% of the patients in the intermediate‐risk group experienced recurrence, suggesting the possibility of low‐intensity ACT. Taken together, through comprehensive biomarker discovery, followed by qRT‐PCR assay‐based clinical biomarker training and validation, we developed a novel GPRSC signature for predicting recurrence in patients with pStage II CRC. The GPRSC signature can provide improved risk stratification compared with conventional criteria, allowing for a more precise prediction of recurrence risk in patients with pStage II CRC.

## Discussion

Despite the extensive efforts of several studies, indications for postoperative ACT in patients with pStage II CRC remain disputed. Owing to the potential treatment‐related toxicity, its impact on patients' quality of life, and the associated economic burden, careful risk stratification is essential to determine the necessity and appropriateness of postoperative ACT for these patients. Currently, several clinical trials have attempted to shorten the duration of postoperative ACT or convert it to monotherapy for pStage II CRC; however, the efficacy of these approaches remains inconclusive [6, 7, 8]. In addition, molecular biomarkers such as ctDNA and immunoscores have emerged as promising tools for recurrence risk assessment and treatment individualization. However, these biomarkers are not widely used in clinical practice. In this study, we conducted comprehensive biomarker discovery and generated the new gene expression‐based predictive signature for recurrence in patients with pStage II CRC using these newly identified biomarkers. Based on these findings and using our novel GPRSC signature and conventional criteria, we developed a novel risk stratification system for postoperative ACT decision‐making in pStage II CRC.

To further assess the clinical utility of our GPRSC signature, we compared our new risk stratification system with the existing NCCN guidelines. According to the NCCN guidelines, patients with MSS tumors and high‐risk clinicopathological features are recommended to receive ACT. However, in our cohort, only 19% of these patients actually experienced recurrence, and treatment decisions were limited to two options: ACT or no ACT. By incorporating the GPRSC signature alongside conventional risk factors, our system enables stratification of patients into three distinct risk groups. The high‐risk group demonstrated a recurrence rate of 41%, indicating a clear need for ACT. In contrast, the intermediate‐risk group exhibited a low recurrence rate of only 6%, suggesting that less intensive chemotherapy or a shortened treatment duration may be appropriate for these patients. This finer stratification provides a more individualized framework for postoperative ACT decision‐making, potentially optimizing treatment intensity according to actual recurrence risk.

Recent significant advances in molecular biology have enabled the identification of novel molecular prognostic and diagnostic biomarkers for cancers, including CRC, and numerous studies have attempted to develop various molecular biomarkers for predicting recurrence in patients with pStage II CRC [16, 27]. However, none of these molecular biomarkers has been applied in clinical practice owing to their potential limitations. This study has several strengths compared with previous studies. First, our biomarker discovery approach was based on systematic genome‐wide transcriptomic profiling comparing recurrent and nonrecurrent pStage II CRC cases, which allowed for the identification of potentially robust biomarkers. Second, this methodology requires only postoperative pathological specimens and utilizes qRT‐PCR‐based assays, making it a practical as well as cost‐effective approach. Third, the biomarkers were validated in an independent patient cohort, thus ensuring the generalizability of our gene expression biomarkers. Fourth, incorporating established clinical risk factors into the GPRSC model enhanced its applicability and acceptability in clinical settings. Fifth, by combining our GPRSC signature combined with MSI status and existing clinical risk factors, we re‐stratified patients with pStage II CRC into three risk groups for those who would benefit from postoperative ACT, offering a new framework that has not been previously utilized in clinical practice.

The 10 candidate genes identified as novel biomarkers in this study have been previously implicated in CRC progression, chemoresistance, or the microenvironment. For example, while *PROK1* promotes lymphangiogenesis and may contribute to tumor growth and lymph node metastasis in CRC [28], *CDH22* is associated with cell adhesion and metastasis [29]. In addition, *SERPINB7* may influence tumor progression through the PI3K/Akt/mTOR signaling pathway in CRC [30]. These known functions support the biological relevance of our predictive model and may help explain its ability to stratify recurrence risk.

MSI‐H is recognized as a prognostic molecular biomarker in patients with pStage II CRC. This molecular feature is associated with a favorable prognosis owing to its high mutational burden, which activates immune responses and promotes effective innate immune surveillance [16]. Therefore, the efficacy of postoperative ACT with fluoropyrimidine monotherapy for pStage II CRC cases with MSI‐H is limited [31], and postoperative ACT is not recommended according to major guidelines, such as the NCCN, ASCO, and ESMO [1, 2, 3]. In the present study, 8.5% of all patients were classified as MSI‐H, which is consistent with previous studies. Furthermore, in line with the favorable prognosis of MSI‐H cases demonstrated in previous studies, no patients with MSI‐H in our clinical cohort experienced recurrence, underscoring the importance of determining the MSI status in therapeutic decision‐making.

The present study had several limitations. First, this study had a retrospective design and was conducted at a single institution with a limited sample size in each cohort. Therefore, prospective multicenter studies with larger patient populations are warranted to validate the predictive utility of the GPRSC signature. Second, the number of MSI‐H cases was relatively small, which may have limited the statistical power of subgroup analyses. Although all eligible cases during the study period were included, further validation in a larger independent cohort is warranted. Ongoing prospective sample collection will enable future re‐evaluation of these findings. Additionally, while the GPRSC signature effectively stratifies patients according to recurrence risk, it currently does not account for individual responses to postoperative ACT, which may limit its ability to predict which patients will benefit the most from these treatments. Future studies should focus on integrating transcriptomic profiles to refine risk assessment further and optimize ACT strategies.

## Conclusions

Through comprehensive genome‐wide biomarker discovery followed by qRT‐PCR‐based biomarker training and validation in clinical surgical specimens, we successfully established a novel and accurate gene expression‐based predictive signature for recurrence in patients with pStage II CRC. Through the integration of MSI status and high‐risk clinical factors for recurrence, this signature can classify patients into three risk groups, identifying those who will benefit from postoperative ACT. This approach may aid in tailored clinical decision‐making for postoperative ACT in patients with pStage II CRC.

## Conflicts of interest

The authors declare no conflicts of interest.

## Author contributions

MO, KO, HK, MT, and YK contributed to the study concept and design. MO, KO, MT, DB, and YK contributed to the provision of samples. MO, KO, SO, and HK contributed to the acquisition of clinical data. MO, KO, and SW contributed to the analysis and interpretation of data and statistical analysis. MO, KO, SW, SO, HK, MT, DB, and YK contributed to the drafting of the manuscript.

## Supporting information


**Fig. S1.** Bar graph with MSI‐H, MSI‐L, and MSS cases in clinical cohorts; MSI‐H, microsatellite instability high; MSI‐L, microsatellite instability low; MSS, microsatellite stable.
**Table S1.** Clinicopathological characteristics of TCGA cohort.
**Table S2.** Primer sequences used in this study and their PCR conditions.
**Table S3.** Details of 52 genes which were significantly differentially expressed in patients with recurrence compared to those without recurrence in TCGA dataset.

## Data Availability

The datasets generated and/or analyzed during the current study are not publicly available due to institutional and collaborative agreements but are available from the corresponding author on reasonable request.
