# Supplementary material for: Identifying gene expression signatures for risk stratification of postoperative adjuvant chemotherapy in colorectal cancer
Source: FEBS Open Bio. 2026 Mar 30;16(9):1813–24. doi: 10.1002/2211-5463.70243 (PMC13398948; doi:10.1002/2211-5463.70243)
Supplement: Supplementary file 1 [file FEB4-16-1813-s001.docx]

**Supporting information**

**Supplementary Figure S1:** Bar graph with MSI-H, MSI-L, and MSS cases in clinical cohorts; MSI-H, microsatellite instability high; MSI-L, microsatellite instability low; MSS, microsatellite stable.

**Supplementary Table S1:** Clinicopathological characteristics of TCGA cohort.

|  |  | TCGA cohort  (n =128) | | |
| --- | --- | --- | --- | --- |
|  |  | n |  | (%) |
| Age (years) | Mean (±SD) | 66 |  | (±13) |
| Sex | Male | 70 |  | (55) |
|  | Female | 58 |  | (45) |
| Tumor location | Right-sided | 60 |  | (47) |
|  | Left-sided | 63 |  | (49) |
|  | Unknown | 5 |  | (4) |
| Tumor depth | T3 | 122 |  | (95) |
|  | T4 | 6 |  | (5) |
| Lymphatic invasion | Positive | 12 |  | (9) |
|  | Negative | 103 |  | (81) |
|  | Unknown | 13 |  | (10) |
| Vascular invasion | Positive | 12 |  | (9) |
|  | Negative | 101 |  | (79) |
|  | Unknown | 15 |  | (12) |
| MSI-H | Positive | 27 |  | (21) |
|  | Negative | 98 |  | (77) |
|  | Unknown | 3 |  | (2) |
| TCGA, The Cancer Genome Atlas; SD, standard deviation; MSI-H, microsatellite instability high. | | | | |

**Supplementary Table S2:** Primer sequences used in this study and their PCR conditions.

| Gene | Sense | Antisense | Size of  the PCR  products  (bp) | Annealing temp  (°C) | GenBank accession  No. |
| --- | --- | --- | --- | --- | --- |
| *CER1* | TTCAGCCAGACTATAACCCACG | AACAGACCCGCATTTCCCAAA | 81 | 60 | NM_005454 |
| *IL29* | AACTGGGAAGGGCTGCCACATT | GGAAGACAGGAGAGCTGCAACT | 134 | 60 | NM_172140 |
| *PROK1* | GTGCCACCCGGGCAG | AGCAAGGACAGGTGTGGTGC | 65 | 60 | NM_032414 |
| *CDH22* | TGTATGTGGGCAAGATCCACT | CTCGTCGATCAGGAAGATGGT | 101 | 58 | NM_021248 |
| *MKRN3* | AGCAGCGGCATTTGGACAA | CGTGCGAATAGCGACAGTTCT | 88 | 60 | NM_005664 |
| *OR10Q1* | CTCCGCACCCCGATGTATTTC | CATCAAGGGTACTACCACGGT | 81 | 60 | NM_001004471 |
| *CALCB* | CACCTGTGTGACTCATCGGC | GGGCACGAAGTTGCTCTTCA | 73 | 60 | NM_000728 |
| *SERPINB7* | TAAGCTCATCTGCTGTAATGGTG | GGCAATTTATGGTTTCGCTCTTG | 93 | 60 | NM_001040147 |
| *SIX3* | CTCCCACACAAGTAGGCAACTG | TCTGCCGAGCCGTGCGTGGG | 148 | 60 | NM_005413 |
| *C17orf78* | TCTTCAGCCTAATCATTGCATCC | GCTGTTCCAGTCGGCAACT | 75 | 60 | NM_173625 |
| *B-Actin* | CCTTTGCCGATCCGCCG | GATATCATCCATGGTGAGCTGG | 56 | 60 | NM_001101 |
| PCR, polymerase chain reaction. | | | | | |

**Supplementary Table S3:** Details of 52 genes which were significantly differentially expressed in patients with recurrence compared to those without recurrence in TCGA dataset.

| **Gene** | **log_2_ FC** | ***P* value** | **FDR** | **baseMean** |
| --- | --- | --- | --- | --- |
| *SLC14A1* | -2.836329202 | 3.08E-10 | 5.76E-06 | 88.03080686 |
| *IGF2* | -3.024005389 | 4.85E-09 | 3.03E-05 | 6021.04336 |
| *CALB1* | -4.091047516 | 8.33E-09 | 3.90E-05 | 62.10987056 |
| *MKRN3* | -4.139846315 | 2.56E-08 | 9.58E-05 | 11.93809592 |
| *SERPINB7* | -3.011334081 | 7.97E-08 | 0.000248957 | 9.558665514 |
| *CPS1* | -2.361004825 | 1.29E-07 | 0.000345732 | 60.5120526 |
| *PRSS21* | -3.129626189 | 2.04E-07 | 0.000476835 | 16.43083358 |
| *HABP2* | -3.042042312 | 3.83E-07 | 0.000797952 | 30.55028615 |
| *CLDN18* | -4.058997726 | 4.64E-07 | 0.000868304 | 335.487824 |
| *C17orf78* | -3.905605213 | 4.08E-06 | 0.00587537 | 5.570080795 |
| *UPK1A* | 2.216026768 | 3.90E-06 | 0.00587537 | 2.804108689 |
| *EYA1* | -2.459275944 | 5.59E-06 | 0.007291681 | 39.10414552 |
| *CNDP1* | -3.368103435 | 5.84E-06 | 0.007291681 | 2.611663116 |
| *UGT1A1* | -2.368941483 | 1.61E-05 | 0.018835998 | 28.72251206 |
| *CALCB* | -3.4566129 | 3.13E-05 | 0.025561049 | 3.835185375 |
| *SULT1E1* | -2.283316266 | 3.01E-05 | 0.025561049 | 9.32154276 |
| *INS-IGF2* | -3.301493248 | 5.28E-05 | 0.036807891 | 9.430054068 |
| *SERPINB3* | -4.437635579 | 5.75E-05 | 0.038437726 | 5.408241658 |
| *CER1* | 2.710303542 | 6.53E-05 | 0.039452831 | 0.769276897 |
| *PPBP* | 2.244373059 | 0.000129939 | 0.063061542 | 135.3737107 |
| *NOTUM* | 2.027705052 | 0.000167077 | 0.076329891 | 1433.22536 |
| *BEST3* | 2.052033464 | 0.000237563 | 0.094676284 | 12.47350912 |
| *CDH7* | -2.426433658 | 0.000259661 | 0.09727432 | 1.395013136 |
| *SLC26A9* | -2.111173725 | 0.000310866 | 0.109864753 | 15.29447392 |
| *AQP5* | 2.453571957 | 0.000325283 | 0.111937197 | 158.9571067 |
| *HAVCR1* | -2.03101603 | 0.000334658 | 0.111937197 | 38.80085869 |
| *OTC* | -2.033305963 | 0.000342008 | 0.112388772 | 64.16635426 |
| *SERPINA7* | -2.156044627 | 0.000504382 | 0.145197105 | 19.80545139 |
| *FAM177B* | -2.027410773 | 0.000488479 | 0.145197105 | 32.84726397 |
| *GP2* | -2.344288751 | 0.000648681 | 0.168756174 | 42.4382454 |
| *SHISA9* | -2.193284828 | 0.000745422 | 0.18262731 | 48.97577428 |
| *UGT2B4* | -3.000312264 | 0.000769763 | 0.18262731 | 2.825671958 |
| *CDH22* | -2.966885228 | 0.000840011 | 0.19667817 | 2.273958302 |
| *FGL1* | -3.458383942 | 0.000955151 | 0.203306117 | 2.598165508 |
| *SIX3* | -2.377003153 | 0.001060022 | 0.213497608 | 3.697128697 |
| *NMUR2* | -2.473175934 | 0.001318652 | 0.236357373 | 8.056356905 |
| *ATP6V0A4* | 2.321654957 | 0.001445926 | 0.240513153 | 4.050466175 |
| *FAM123C* | -2.030185267 | 0.001539254 | 0.244336957 | 2.700592474 |
| *PROK1* | 2.352621575 | 0.002091402 | 0.277196628 | 0.777039998 |
| *SLCO1A2* | -2.369688513 | 0.00240216 | 0.290289363 | 2.353841335 |
| *CYP1A1* | -2.83264073 | 0.002911677 | 0.334592791 | 2.837156711 |
| *BPIL1* | 4.158230032 | 0.004300892 | 0.402800008 | 1.192632266 |
| *IL29* | 2.230440965 | 0.004611043 | 0.41558894 | 0.494060772 |
| *KIR2DL1* | -2.007694039 | 0.004818251 | 0.425710689 | 1.132386606 |
| *KRT31* | -2.660740287 | 0.005309577 | 0.45068239 | 1.4219236 |
| *SERPINB4* | -2.609490102 | 0.00540782 | 0.45068239 | 1.377961633 |
| *RGR* | 2.111515589 | 0.005708367 | 0.458898795 | 7.854430516 |
| *GATA4* | -3.097424009 | 0.00663893 | 0.480130483 | 28.59856098 |
| *LMX1A* | -2.006595796 | 0.007423346 | 0.51120108 | 1.056302173 |
| *GATA5* | -2.191165961 | 0.00753781 | 0.5152946 | 2.29498085 |
| *OR10Q1* | -2.018796086 | 0.008082659 | 0.536866231 | 0.75385196 |
| *ITLN2* | -2.087334428 | 0.009712519 | 0.584325284 | 6.372234742 |
| FC, fold-change; FDR, false discovery rate. | | | | |
