# Supplementary material for: Identifying gene expression signatures for risk stratification of postoperative adjuvant chemotherapy in colorectal cancer
Source: FEBS Open Bio. 2026 Mar 30:10.1002/2211-5463.70243. Online ahead of print. doi: 10.1002/2211-5463.70243 (PMC13398948; doi:10.1002/2211-5463.70243)
Supplement: Supplementary file 1 — Fig. S1. Bar graph with MSI‐H, MSI‐L, and MSS cases in clinical cohorts; MSI‐H, microsatellite instability high; MSI‐L, microsatellite instability low; MSS, microsatellite stable. Table S1. Clinicopathological characteristics of TCGA cohort. Table S2. Primer sequences used in this study and their PCR conditions. Table S3. Details of 52 genes which were significantly differentially expressed in patients with recurrence compared to those without recurrence in TCGA dataset. [file FEB4-9999-0-s001.docx]

**Supporting information**

**Supplementary Figure S1:** Bar graph with MSI-H, MSI-L, and MSS cases in clinical cohorts; MSI-H, microsatellite instability high; MSI-L, microsatellite instability low; MSS, microsatellite stable.

**
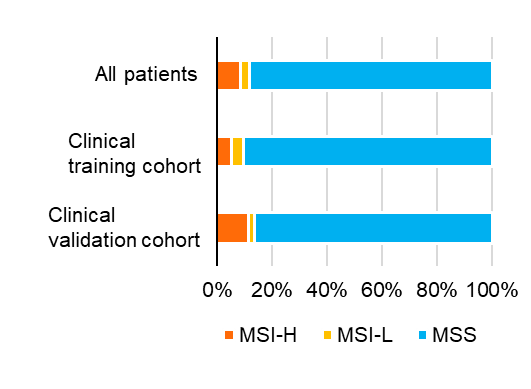
**

**Supplementary Table S1:** Clinicopathological characteristics of TCGA cohort.

|  | | | | |
| --- | --- | --- | --- | --- |
|  |  | TCGA cohort  (n =128) | | |
|  |  | n |  | (%) |
| Age (years) | Mean (±SD) | 66 |  | (±13) |
| Sex | Male | 70 |  | (55) |
|  | Female | 58 |  | (45) |
| Tumor location | Right-sided | 60 |  | (47) |
|  | Left-sided | 63 |  | (49) |
|  | Unknown | 5 |  | (4) |
| Tumor depth | T3 | 122 |  | (95) |
|  | T4 | 6 |  | (5) |
| Lymphatic invasion | Positive | 12 |  | (9) |
|  | Negative | 103 |  | (81) |
|  | Unknown | 13 |  | (10) |
| Vascular invasion | Positive | 12 |  | (9) |
|  | Negative | 101 |  | (79) |
|  | Unknown | 15 |  | (12) |
| MSI-H | Positive | 27 |  | (21) |
|  | Negative | 98 |  | (77) |
|  | Unknown | 3 |  | (2) |
| TCGA, The Cancer Genome Atlas; SD, standard deviation; MSI-H, microsatellite instability high. | | | | |

**Supplementary Table S2:** Primer sequences used in this study and their PCR conditions.

|  | | | | | |
| --- | --- | --- | --- | --- | --- |
| Gene | Sense | Antisense | Size of  the PCR  products  (bp) | Annealing temp  (°C) | GenBank accession  No. |
| *CER1* | TTCAGCCAGACTATAACCCACG | AACAGACCCGCATTTCCCAAA | 81 | 60 | NM_005454 |
| *IL29* | AACTGGGAAGGGCTGCCACATT | GGAAGACAGGAGAGCTGCAACT | 134 | 60 | NM_172140 |
| *PROK1* | GTGCCACCCGGGCAG | AGCAAGGACAGGTGTGGTGC | 65 | 60 | NM_032414 |
| *CDH22* | TGTATGTGGGCAAGATCCACT | CTCGTCGATCAGGAAGATGGT | 101 | 58 | NM_021248 |
| *MKRN3* | AGCAGCGGCATTTGGACAA | CGTGCGAATAGCGACAGTTCT | 88 | 60 | NM_005664 |
| *OR10Q1* | CTCCGCACCCCGATGTATTTC | CATCAAGGGTACTACCACGGT | 81 | 60 | NM_001004471 |
| *CALCB* | CACCTGTGTGACTCATCGGC | GGGCACGAAGTTGCTCTTCA | 73 | 60 | NM_000728 |
| *SERPINB7* | TAAGCTCATCTGCTGTAATGGTG | GGCAATTTATGGTTTCGCTCTTG | 93 | 60 | NM_001040147 |
| *SIX3* | CTCCCACACAAGTAGGCAACTG | TCTGCCGAGCCGTGCGTGGG | 148 | 60 | NM_005413 |
| *C17orf78* | TCTTCAGCCTAATCATTGCATCC | GCTGTTCCAGTCGGCAACT | 75 | 60 | NM_173625 |
| *B-Actin* | CCTTTGCCGATCCGCCG | GATATCATCCATGGTGAGCTGG | 56 | 60 | NM_001101 |
| PCR, polymerase chain reaction. | | | | | |

**Supplementary Table S3:** Details of 52 genes which were significantly differentially expressed in patients with recurrence compared to those without recurrence in TCGA dataset.

|  | | | | |
| --- | --- | --- | --- | --- |
| **Gene** | **log_2_ FC** | ***P* value** | **FDR** | **baseMean** |
| *SLC14A1* | -2.836329202 | 3.08E-10 | 5.76E-06 | 88.03080686 |
| *IGF2* | -3.024005389 | 4.85E-09 | 3.03E-05 | 6021.04336 |
| *CALB1* | -4.091047516 | 8.33E-09 | 3.90E-05 | 62.10987056 |
| *MKRN3* | -4.139846315 | 2.56E-08 | 9.58E-05 | 11.93809592 |
| *SERPINB7* | -3.011334081 | 7.97E-08 | 0.000248957 | 9.558665514 |
| *CPS1* | -2.361004825 | 1.29E-07 | 0.000345732 | 60.5120526 |
| *PRSS21* | -3.129626189 | 2.04E-07 | 0.000476835 | 16.43083358 |
| *HABP2* | -3.042042312 | 3.83E-07 | 0.000797952 | 30.55028615 |
| *CLDN18* | -4.058997726 | 4.64E-07 | 0.000868304 | 335.487824 |
| *C17orf78* | -3.905605213 | 4.08E-06 | 0.00587537 | 5.570080795 |
| *UPK1A* | 2.216026768 | 3.90E-06 | 0.00587537 | 2.804108689 |
| *EYA1* | -2.459275944 | 5.59E-06 | 0.007291681 | 39.10414552 |
| *CNDP1* | -3.368103435 | 5.84E-06 | 0.007291681 | 2.611663116 |
| *UGT1A1* | -2.368941483 | 1.61E-05 | 0.018835998 | 28.72251206 |
| *CALCB* | -3.4566129 | 3.13E-05 | 0.025561049 | 3.835185375 |
| *SULT1E1* | -2.283316266 | 3.01E-05 | 0.025561049 | 9.32154276 |
| *INS-IGF2* | -3.301493248 | 5.28E-05 | 0.036807891 | 9.430054068 |
| *SERPINB3* | -4.437635579 | 5.75E-05 | 0.038437726 | 5.408241658 |
| *CER1* | 2.710303542 | 6.53E-05 | 0.039452831 | 0.769276897 |
| *PPBP* | 2.244373059 | 0.000129939 | 0.063061542 | 135.3737107 |
| *NOTUM* | 2.027705052 | 0.000167077 | 0.076329891 | 1433.22536 |
| *BEST3* | 2.052033464 | 0.000237563 | 0.094676284 | 12.47350912 |
| *CDH7* | -2.426433658 | 0.000259661 | 0.09727432 | 1.395013136 |
| *SLC26A9* | -2.111173725 | 0.000310866 | 0.109864753 | 15.29447392 |
| *AQP5* | 2.453571957 | 0.000325283 | 0.111937197 | 158.9571067 |
| *HAVCR1* | -2.03101603 | 0.000334658 | 0.111937197 | 38.80085869 |
| *OTC* | -2.033305963 | 0.000342008 | 0.112388772 | 64.16635426 |
| *SERPINA7* | -2.156044627 | 0.000504382 | 0.145197105 | 19.80545139 |
| *FAM177B* | -2.027410773 | 0.000488479 | 0.145197105 | 32.84726397 |
| *GP2* | -2.344288751 | 0.000648681 | 0.168756174 | 42.4382454 |
| *SHISA9* | -2.193284828 | 0.000745422 | 0.18262731 | 48.97577428 |
| *UGT2B4* | -3.000312264 | 0.000769763 | 0.18262731 | 2.825671958 |
| *CDH22* | -2.966885228 | 0.000840011 | 0.19667817 | 2.273958302 |
| *FGL1* | -3.458383942 | 0.000955151 | 0.203306117 | 2.598165508 |
| *SIX3* | -2.377003153 | 0.001060022 | 0.213497608 | 3.697128697 |
| *NMUR2* | -2.473175934 | 0.001318652 | 0.236357373 | 8.056356905 |
| *ATP6V0A4* | 2.321654957 | 0.001445926 | 0.240513153 | 4.050466175 |
| *FAM123C* | -2.030185267 | 0.001539254 | 0.244336957 | 2.700592474 |
| *PROK1* | 2.352621575 | 0.002091402 | 0.277196628 | 0.777039998 |
| *SLCO1A2* | -2.369688513 | 0.00240216 | 0.290289363 | 2.353841335 |
| *CYP1A1* | -2.83264073 | 0.002911677 | 0.334592791 | 2.837156711 |
| *BPIL1* | 4.158230032 | 0.004300892 | 0.402800008 | 1.192632266 |
| *IL29* | 2.230440965 | 0.004611043 | 0.41558894 | 0.494060772 |
| *KIR2DL1* | -2.007694039 | 0.004818251 | 0.425710689 | 1.132386606 |
| *KRT31* | -2.660740287 | 0.005309577 | 0.45068239 | 1.4219236 |
| *SERPINB4* | -2.609490102 | 0.00540782 | 0.45068239 | 1.377961633 |
| *RGR* | 2.111515589 | 0.005708367 | 0.458898795 | 7.854430516 |
| *GATA4* | -3.097424009 | 0.00663893 | 0.480130483 | 28.59856098 |
| *LMX1A* | -2.006595796 | 0.007423346 | 0.51120108 | 1.056302173 |
| *GATA5* | -2.191165961 | 0.00753781 | 0.5152946 | 2.29498085 |
| *OR10Q1* | -2.018796086 | 0.008082659 | 0.536866231 | 0.75385196 |
| *ITLN2* | -2.087334428 | 0.009712519 | 0.584325284 | 6.372234742 |
| FC, fold-change; FDR, false discovery rate. | | | | |
